# Expression of HIF-1α is related to a poor prognosis and tamoxifen resistance in contralateral breast cancer

**DOI:** 10.1371/journal.pone.0226150

**Published:** 2019-12-10

**Authors:** Annika Jögi, Anna Ehinger, Linda Hartman, Sara Alkner

**Affiliations:** 1 Department of Laboratory Medicine, Translational Cancer Research, Lund University Cancer Center at Medicon Village, Lund University, Lund, Sweden; 2 Lund University, Skåne University Hospital, Department of Clinical Sciences Lund, Division of Oncology and Pathology, Lund, Sweden; 3 Lund University, Department of Clinical Genetics and Pathology, Medical Service, Regional Laboratories, Lund, Sweden; Turun Yliopisto, FINLAND

## Abstract

**Background:**

Adjuvant endocrine treatment improves survival after estrogen receptor (ER) positive breast cancer. Recurrences occur, and most patients with metastatic breast cancer develop treatment resistance and incurable disease. An influential factor in relation to endocrine treatment resistance is tumor hypoxia and the hypoxia inducible transcription factors (HIFs). Poor perfusion makes tumors hypoxic and induces the HIFs, which promote cell survival. We previously showed that hypoxic breast cancer cells are tamoxifen-resistant, and that HIF-inhibition restored tamoxifen-sensitivity. We found that HIF-induced tamoxifen-resistance involve cross-talk with epithelial growth factor receptor (EGFR), which itself is linked to tamoxifen resistance. Contralateral breast cancer (CBC), *i*.*e*. development of a second breast cancer in the contralateral breast despite adjuvant tamoxifen treatment is in essence a human *in vivo*-model for tamoxifen-resistance that we explore here to find molecular pathways of tamoxifen-resistance.

**Methods:**

We constructed a tissue-microarray including tumor-tissue from a large well-defined cohort of CBC-patients, a proportion of which got their second breast cancer despite ongoing adjuvant therapy. Using immunohistochemistry >500 patients were evaluable for HIF-1α and EGFR in both tumors, and correlations to treatment, patient outcome, prognostic and predictive factors were analyzed.

**Results:**

We found an increased proportion of HIF-1α-positive tumors in tamoxifen-resistant (CBC during adjuvant tamoxifen) compared to naïve tumors (CBC without prior tamoxifen). Tumor HIF-1α-positivity correlated to increased breast cancer mortality, and negative prognostic factors including low age at diagnosis and ER-negativity. There was a covariance of HIF-1α- and EGFR-expression and also EGFR-expression correlated to poor prognosis.

**Conclusions:**

The increased percentage of HIF-1α-positive tumors formed during adjuvant tamoxifen suggests a role for HIF-1α in escaping tamoxifen’s restraining effects on breast cancer. Implicating a potential benefit of HIF-inhibitors in targeting breast cancers resistant to endocrine therapy.

## Introduction

The majority of breast cancers express estrogen receptor α (ERα) [1], and adjuvant endocrine therapy significantly improves patient-survival. Tamoxifen is the most common adjuvant endocrine treatment in premenopausal women and reduces recurrences with about 50% [2]. It is also widely used for treatment of ERα-positive generalized breast cancer. Despite therapy many patients receiving adjuvant treatment, and practically all with metastatic disease, eventually relapse and die from their cancer. Resistance can be intrinsic (*de novo* resistance), or arise during treatment (acquired resistance) [3]. Here we employ a novel approach to study endocrine therapy escape mechanisms *in vivo* in breast cancer patients by analyzing metachronous contralateral breast cancer (CBC), *i*.*e*. development of a second breast cancer (BC2) in the contralateral breast despite on-going adjuvant tamoxifen treatment given for the first tumor (BC1), and therefore resistant to this treatment. We have assembled a unique tissue microarray (TMA) including >700 patients from a well-defined population-based cohort with metachronously developed CBC. Detailed information on outcome, epidemiological factors, treatment, and tumor clinical and pathological variables is available for each patient. CBC developed after prior endocrine therapy was to a larger extent ERα-negative. Prior endocrine therapy, chemotherapy, and radiotherapy were all associated with a worse prognosis once diagnosed with CBC [4–6].

Breast cancers, like most tumors, are hypoxic due to insufficient perfusion. In response to hypoxia the hypoxia-inducible transcription factor alpha-subunits, HIF-1α and HIF-2α, are accumulated and activated [7–9]. The alpha-subunit dimerizes with HIF-β forming a transcription factor-complex that translocates to the nucleus where it binds to hypoxia-responsive-elements (HRE) in the genome. The six-nucleotide HRE core-sequence is identical for HIF-1 and HIF-2, hence their transcriptomes largely overlap [10]. The hypoxic transcriptome includes genes involved in angiogenesis, glycolysis, cell survival and proliferation, mechanisms that can contribute to cancer progression [11]. Hypoxia and high protein levels of HIF-1α and HIF-2α correlate with poor prognosis in breast cancer patients [12–14]. We previously showed that ERα-positive breast cancer cells (MCF-7, CAMA, and T47D) grown under hypoxic conditions were resistant to antiestrogens (tamoxifen and fulvestrant), while they were sensitive to treatment at normoxia, and treating the resistant hypoxic cancer cells with a HIF-inhibitor (FM19G11) restored antiestrogen sensitivity [15]. The hypoxia-induced antiestrogen-resistance was conveyed via epithelial growth factor receptor (EGFR) and bilateral cross-talk between, at least, HIF-2α and EGFR occurred in antiestrogen-resistant cell lines [15]. Additional researchers have implicated EGFR in antiestrogen resistance [16], and HIF-1α seem to covariate with EGFR-expression in breast cancer [17].

The aim of the current paper was to test if tumor hypoxia and HIF-1α, the most robustly hypoxia-induced HIF, contribute to endocrine treatment resistance in breast cancer patients. Addressing possible signaling pathways, we also tested if HIF-1α and EGFR-expression correlate. For this purpose, we analyzed CBCs formed during ongoing endocrine treatment since it is a human *in vivo*-model of treatment resistance. We show here that an increased proportion of tamoxifen-resistant CBCs, *i*.*e*. tumors developed during adjuvant tamoxifen treatment, were HIF-1α positive and that HIF-1α-positivity in the tumors led to increased breast cancer mortality (BCM). We also confirm a strong correlation between HIF-1α and EGFR, opening for new possible treatment strategies to overcome tamoxifen resistance.

## Materials and methods

### Aim

We aimed to test whether tumor hypoxia and HIF-1α contribute to endocrine treatment resistance in a human breast cancer material where a second breast cancer developed despite ongoing tamoxifen treatment, and hence show tamoxifen resistance. We further aim to investigate if HIF-1α and EGFR-expression correlate, and how these proteins affect prognosis/breast cancer mortality.

### Patient Cohort and Tissue Microarray (TMA)

**Fig 1:**
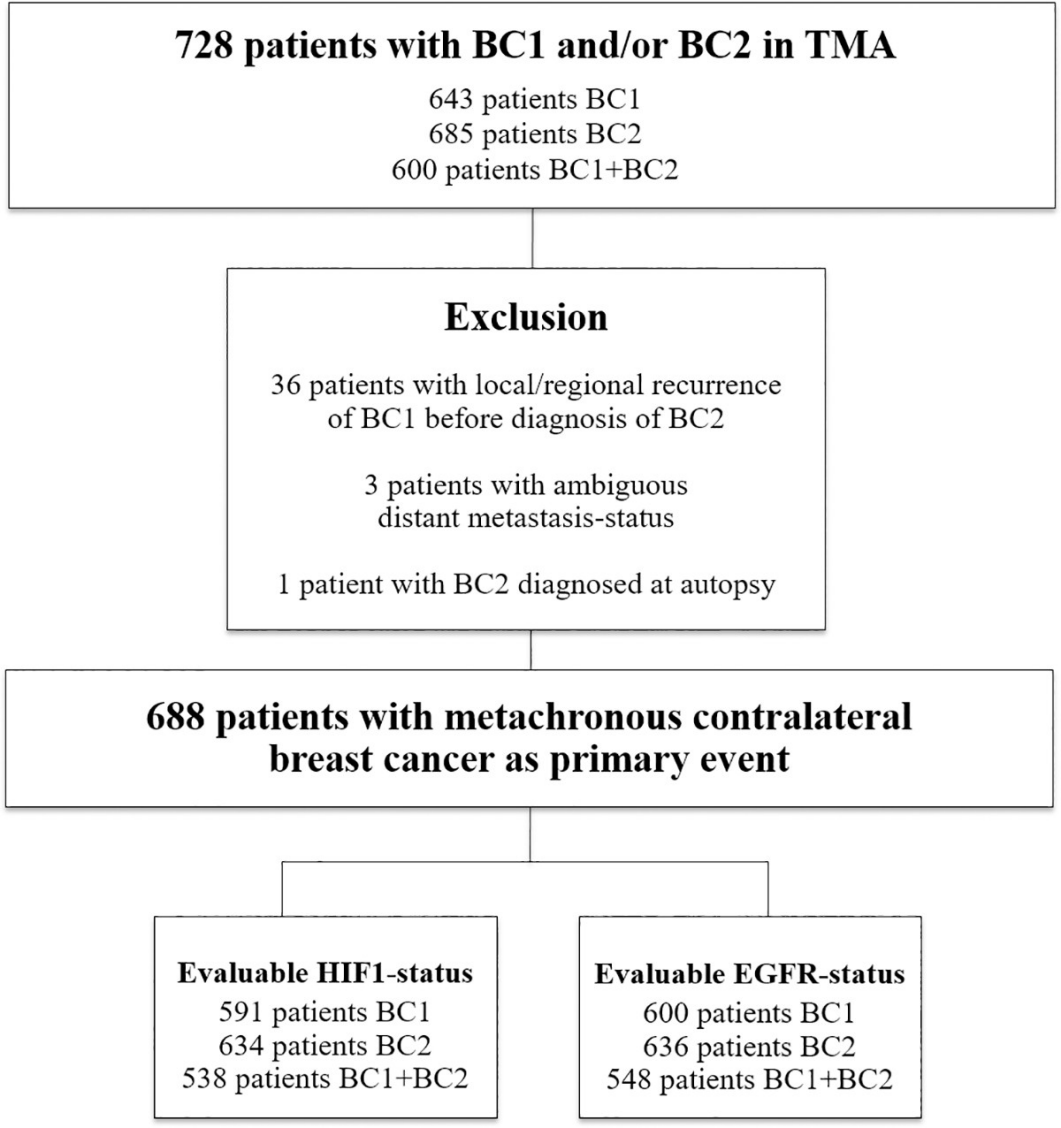
Flow-chart of inclusion vs. exclusion in the study cohort. In analysis, 36 patients with a local/regional recurrence of BC1 before diagnosis of BC2 were excluded in order not to confuse the results by eventual treatment given for the recurrence. We also excluded 3 patients with ambiguous distant metastasis-status and 1 patient with BC2 diagnosed at autopsy.

Inclusion criteria, data abstraction, and TMA-construction have been previously described [5, 18]. Briefly, all patients within the Southern Swedish Healthcare Region with two breast cancers in the Swedish Cancer Registry (BC2 diagnosed 1977–2007) were included. Clinical data were abstracted from individual charts and formalin-fixed paraffin-embedded (FFPE) tissue collected. We focused on metachronous CBC (≥3 months between tumors), excluding patients with synchronous CBC, distant metastasis or another malignancy diagnosed before BC2, or with BC2 found only in the axilla. For the 764 patients fulfilling these criteria, paraffin-blocks were available for 643 BC1 and 685 BC2 (both tumors in 600 cases), giving a total of 728 patients included in the TMA (Fig 1). From representative areas of the invasive breast cancers, at least two tissue-core-biopsies (diameter 1.0 mm) from each tumor were punched out and mounted into the recipient block using a tissue-array-machine (Beecher Instruments, USA). After exclusion according to predefined criteria (Fig 1) 688 patients remained for HIF-1α- and EGFR-evaluation (Fig 1). Prognosis and hormone receptor status in relation to both tumors and treatment was previously presented [18, 19].

Since all patients with another malignancy before CBC were excluded, no patients had received chemotherapy or endocrine therapy prior to diagnosis of their first breast cancer. However, 6 patients had received previous radiotherapy towards the chest due to benign afflictions. Chemotherapy was given to 66 patients for BC1 and 50 patients for BC2. Neoadjuvant treatment was given to 5 of the 62 patients that received chemotherapy for BC1, and to 2 of the 47 patients that received chemotherapy for BC2.

Endocrine treatment was given to 159 of 681 patients for BC1 (missing data for 7 patients). Endocrine treatment used was tamoxifen only for 141 patients. Fourteen patients were treated with oophorectomy, 1 patient received oophorectomy + tamoxifen, 1 patient received tamoxifen followed by an aromatase inhibitor, and 2 patients received androgens. Of the patients that received endocrine treatment for BC1 86% was ER-positive, and 14% ER-negative. However, only 25% of patients with an ER-positive BC1 received endocrine treatment, since this was not clinical routine at the time of their diagnosis (most patients had their BC1 before 1996, Table 1). The corresponding numbers for BC2 were that endocrine treatment was given to 274 of 683 patients (missing data for 5 patients). For 225 patients endocrine treatment used was tamoxifen only, for 23 aromatase inhibitor only, for 16 a combination of tamoxifen and aromatase inhibitor, for 3 patients oophorectomy only, for 2 oophorectomy + tamoxifen/aromatase inhibitor, for 1 megestrolacetat, and for 1 megestrolacetat + tamoxifen (missing data for 3 patients). Of the patients that received endocrine treatment for BC2 87% were ER-positive and 13% ER-negative. In total, 44% of patients with an ER-positive BC2 received endocrine treatment. Patients were considered to have used systemic endocrine treatment only if treatment had continued for more than 3 months.

**Table 1:** Patient and tumor characteristics in relation to HIF and EGFR-status of both tumors.

N = 688	First breast cancer, N (%)						Second breast cancer, N (%)					
HIF1/EGFR missing for 97/88 BC1, 54/52 BC2	**HIF1-negative**	**HIF1-positive**	**P-value**^**a**^	**EGFR-negative**	**EGFR-positive**	**P-value**^**a**^	**HIF1-negative**	**HIF1-positive**	**P-value**^**a**^	**EGFR-negative**	**EGFR-positive**	**P-value**^**a**^
	**N = 522 (88%)**	**N = 69 (12%)**		**N = 520 (87%)**	**N = 80 (13%)**		**N = 523 (82%)**	**N = 111 (18%)**		**N = 570 (90%)**	**N = 66** **(10%)**	
**Date of diagnosis**			0.6			0.001			0.2			0.9
<1977	82 (92%)	7 (8%)		68 (75%)	23 (25%)		0	0		0	0	
1977–1986	158 (87%)	23 (13%)		159 (87%)	24 (13%)		102 (86%)	16 (14%)		107 (90%)	12 (10%)	
1987–1996	204 (87%)	30 (13%)		214 (90%)	25 (10%)		181 (82%)	39 (18%)		199 (90%)	23 (10%)	
1997–2007	78 (90%)	9 (10%)		79 (91%)	8 (9%)		240 (81%)	56 (19%)		264 (89%)	31 (11%)	
**Age at diagnosis**			0.009			<0.001			0.03			<0.001
<50 years	140 (83%)	29 (17%)		139 (79%)	37 (21%)		52 (73%)	19 (27%)		54 (76%)	17 (24%)	
≥50 years	382 (91%)	40 (9%)		381 (90%)	43 (10%)		471 (84%)	92 (16%)		516 (91%)	49 (9%)	
**Node status**			0.9			0.04			0.002			0.5
N0	323 (88%)	43 (12%)		317 (85%)	57 (15%)		289 (85)	51 (15%)		304 (90%)	35 (10%)	
N+	165 (88%)	23 (12%)		172 (91%)	17 (9%)		141 (74)	49 (26%)		167 (88%)	23 (12%)	
If N+ Median (range)	3 (1–33)	2 (1–12)		2.5 (1–33)	2.5 (1–10)		2 (1–23)	2 (1–21)		2 (1–23)	4 (1–11)	
*Missing*	*34*	*3*		*31*	*6*		*93*	*11*		*99*	*8*	
**Size**			0.4			0.6			0.7			0.01
≤20 mm	312 (89%)	38 (11%)		314 (88%)	43 (12%)		366 (83%)	75 (17%)		406 (91%)	38 (9%)	
>20 mm	177 (87%)	27 (13%)		176 (86%)	28 (14%)		143 (82%)	32 (18%)		147 (84%)	27 (16%)	
Median (range)	18 (1–100)	20 (1–70)		17 (1–100)	20 (1–70)		15 (1–110)	17 (1–80)		15 (1–110)	20 (1–80)	
*Missing*	*33*	*4*		*30*	*9*		*14*	*4*		*17*	*1*	
**Stage**			0.4			0.3			0.008			0.1
I	233 (89%)	29 (11%)		232 (87%)	36 (13%)		234 (86%)	39 (14%)		247 (90%)	26 (10%)	
II	163 (88%)	22 (12%)		162 (87%)	24 (13%)		123 (77%)	36 (23%)		142 (90%)	16 (10%)	
III	55 (85%)	10 (15%)		60 (92%)	5 (8%)		62 (75%)	21 (25%)		69 (83%)	14 (17%)	
*Missing*	*71*	*8*		*66*	*15*		*104*	*15*		*112*	*10*	
**ER-status**			<0.001			<0.001			<0.001			<0.001
<10%	75 (76%)	24 (24%)		49 (51%)	48 (49%)		54 (52%)	50 (48%)		54 (52%)	50 (48%)	
≥10%	442 (91%)	44 (9%)		464 (94%)	29 (6%)		463 (89%)	57 (11%)		507 (97%)	14 (3%)	
*Missing*	*5*	*1*		*7*	*3*		*6*	*4*		*9*	*2*	
**PR-status**			<0.001			<0.001			<0.001			<0.001
<10%	132 (80%)	33 (20%)		110 (67%)	53 (43%)		145 (69%)	65 (31%)		158 (75%)	52 (25%)	
≥10%	385 (92%)	35 (8%)		403 (94%)	24 (6%)		367 (90%)	42 (10%)		399 (97%)	11 (3%)	
*Missing*	*5*	*1*		*7*	*3*		*11*	*4*		*13*	*3*	
**HER2-status**			0.005			0.07			<0.001			0.07
Negative (0 to 2+)	482 (90%)	56 (10%)		476 (88%)	68 (13%)		495 (85%)	85 (15%)		526 (90%)	56 (10%)	
Positive (3+)	30 (75%)	10 (25%)		31 (78%)	9 (23%)		18 (49%)	19 (51%)		30 (81%)	7 (19%)	
*Missing*	*10*	*3*		*13*	*3*		*10*	*7*		*14*	*3*	
**Ki67**			<0.001			<0.001			<0.001			<0.001
≤20	440 (93%)	34 (7%)		440 (92%)	39 (8%)		442 (90%)	51 (10%)		469 (95%)	26 (5%)	
>20	72 (69%)	32 (31%)		67 (64%)	37 (36%)		65 (55%)	53 (45%)		82 (69%)	36 (31%)	
*Missing*	*10*	*3*		*13*	*4*		*16*	*7*		*19*	*4*	
**EGFR**			<0.001						<0.001			
Negative	467 (91%)	45 (9%)		*-*	*-*		493 (87%)	73 (13%)		*-*	*-*	
Positive	54 (70%)	23 (30%)		*-*	*-*		29 (44%)	37 (56%)		*-*	*-*	
*Missing*	*1*	*1*					*1*	*1*				
**Subtype**			<0.001			<0.001			<0.001			<0.001
Luminal A-like	330 (93%)	23 (7%)		338 (94%)	20 (6%)		314 (94%)	21 (6%)		331 (98%)	6 (2%)	
Luminal B-like HER2-	97 (87%)	15 (13%)		105 (94%)	7 (6%)		127 (84%)	25 (16%)		147 (97%)	5 (3%)	
Luminal B-like HER2+	12 (71%)	5 (29%)		16 (94%)	1 (6%)		10 (53%)	9 (47%)		17 (89%)	2 (11%)	
HER2+	16 (76%)	5 (24%)		13 (62%)	8 (38%)		7 (44%)	9 (56%)		11 (69%)	5 (31%)	
Triple Negative	45 (71%)	18 (29%)		24 (38%)	39 (62%)		38 (51%)	37 (49%)		32 (43%)	43 (57%)	
*Missing*	*22*	*3*		*24*	*5*		*27*	*10*		*32*	*5*	
**Time-interval BC1-BC2**			0.04			0.8			0.2			0.1
<5 year	226 (85%)	39 (15%)		231(86%)	37 (14%)		215 (80%)	53 (20%)		235 (87%)	34 (13%)	
≥5 years	296 (91%)	30 (9%)		289 (87%)	43 (13%)		308 (84%)	58 (16%)		335 (91%)	32 (9%)	

**Abbreviations: BC1** *first breast cancer* **BC2** *second breast cancer*, **EGFR** *Epidermial growth factor receptor*, **ER** *estrogen receptor*, **HIF** *Hypoxia-inducible factor*, **N+** *lymph-node metastases*, **N0** *no lymph-node metastases*, **N** *number*, **node status** *lymph-node status*, **PR** *progesterone receptor*.

^a^ χ^2^-test except for date of diagnosis and stage where a χ^2^-test for trend was used.

In 60 patients their BC2 was diagnosed while under tamoxifen for BC1. In analyses below regarding tamoxifen’s effect on characteristics of BC2, these patients are compared with patients with no prior tamoxifen before BC2. Patient and tumor characteristic in relation to HIF-1α and EGFR are described in Table 1.

### Cells and cell culture

The MCF-7/S0.5 (MCF-7, parental cells) and the antiestrogen resistant strains that were derived from it, MCF-7/TAM^R^-1 (TAMR1), MCF-7/TAM^R^-7 (TAMR7), MCF-7/182^R^-6 (182R6) and MCF-7/164^R^-7 (164R7) were a kind gift from dr Anne Lykkesfeldt, Danish Cancer Society Research Center. MCF-7/S0.5 was established by stepwise reduction of the serum concentration from 5% to 0.5% [20]. The tamoxifen-resistant TAMR-1 and -7 and the fulvestrant-resistant 164R6 and 184R7 cells were established by long term selection with 1μM tamoxifen, 0.1 μM ICI 182,780 and 0.1 μM ICI 164,384, respectively [21, 22]. MCF-7 cell line authenticity was tested and positively confirmed (DSMZ, Germany). All cells were grown in standard DMEM/F12 medium (Thermo Fisher Scientific, MA) with 1% FCS (Biosera, MO), penicillin and streptomycin (100 units/ml, Hyclone, GE Healthcare, UT) and insulin (100 units/ml, Actrapid, Novo Nordisk, Denmark). The cells were routinely cultured at 37°C, 5% CO_2_, and air oxygen levels, kept at low passage numbers, and checked for *Mycoplasma* on a monthly basis with consistently negative results. Antiestrogen-resistant cells were maintained in their respective antiestrogen until 1–2 weeks prior to experimental use. Hypoxic cell culture experiments were performed in Don Whitley Hypoxystation (Don Whitley Scientific, Shipley, UK) under identical culture conditions except for oxygen levels.

### Immunoblotting

Whole cell lysates (40–80 μg protein in RIPA buffer with Complete, Roche, Switzerland) were electrophoretically separated (7.5% Mini TGX gel, BioRad Laboratories CA, according to manufacturers instructions). Protein detection was performed using antibodies against HIF-1α (Becton Dickinson, NJ), ERα (Cell Signaling Technologies, MA), actin (MP Biomedicals, CA) and SDHA (Ab14715, Abcam).

### Immunohistochemistry

All immunohistochemistry (IHC) was performed on FFPE 4 μm sections in an Autostainer-*Plus* (Dako) according to manufacturer’s protocol. IHC for Ki67 (M7240-Dako), ERα (RM-9101 ThermoScientific), and progesterone receptor (PR) (M3569-Dako) were previously descried [23]. For HER2 the Ventana Benchmark system was used (Ventana 790–2991). An experienced clinical pathologist (AE) reevaluated expression of ERα, PR, HER2, and Ki67 in the tumor samples. In line with Swedish clinical standard at this time tumors with ≥10% stained nuclei were considered ERα-/PR-positive. Tumor cores with HER2 IHC-signal of 3+ were considered HER2-positive, *in situ* hybridization was not performed. Samples with Ki67-expression in >20% of cell nuclei were considered Ki67-high.

For HIF-1α IHC monoclonal antibody BD610959 (Becton Dickinson) diluted 1:50 was employed. EGFR-expression (M7239 dilution 1:25, Dako) was analyzed according to the EGFR pharmDX^TM^ Interpretation Manual (Dako), an FDA-approved assay intended as an aid in finding patients eligible for EGFR-targeting therapy. Two physicians blinded for clinical/tumor-characteristics (AJ, SA) independently assessed IHC-staining for EGFR and HIF-1α. For HIF-1α each sample was semi-quantitatively scored from 0–3 for percentage of stained cells and staining intensity. Proportion score 0 represented no positive cells, 1: 1–10%, 2: 11–50%, and 3: 51–100%. Intensity 0 represented negative, 1 weak, 2 moderate, and 3 intense IHC-signal. In case of discrepant staining between the two cores from the same patient, the highest score was used. Cases with differing HIF-1α/EGFR-positivity results between viewers were reexamined independently by an experienced viewer (KL). Surrogate definitions of intrinsic subtypes were defined using IHC-annotated biomarker according to the St Gallen-guidelines [24].

### Statistical analysis

Survival-data and cause of death was retrieved from the Swedish National Board of Health and Welfare (March 2014), and BCM chosen as primary end-point. BCM was defined as breast cancer death or death after metastasis and was measured from CBC-diagnosis. For statistical calculations, the software package Stata 13.1 (StataCorp, USA) was used. Associations between HIF/EGFR-values/prior tamoxifen and patient/tumor-characteristics were evaluated with the χ^2^-test or the χ^2^-test for trend, while general comparisons between groups of BC1 and BC2 were done with McNemar’s test. Prognosis after BC2 was summarized graphically as cumulative BCM. Cause-specific Cox-regression, treating competing events as censoring, was used to estimate hazard ratios (HR). To assess whether the effect of a factor differed in different subgroups, Cox models with a term for interaction were used. Assumptions of proportional hazards were checked graphically. To summarize variability in estimated effects 95% confidence intervals (CI), corresponding to a p-value threshold of 0.05, were used.

Approximately 90% of patients with endocrine therapy for BC1 received tamoxifen (141/159). Patients with other endocrine treatment than tamoxifen for BC1 were excluded from analyses regarding tamoxifen. Other prior adjuvant treatment did not significantly differ between patients with *vs*. without tamoxifen for BC1 (radiotherapy 61% *vs*. 63%, chemotherapy 6% *vs*. 11%). In 60 patients their BC2 was diagnosed while under tamoxifen for BC1. In analyses below regarding tamoxifen’s effect on characteristics of BC2, these patients are compared with patients with no prior tamoxifen before BC2.

## Results

### HIF-1α-positivity correlated to negative prognostic factors

**Fig 2:**
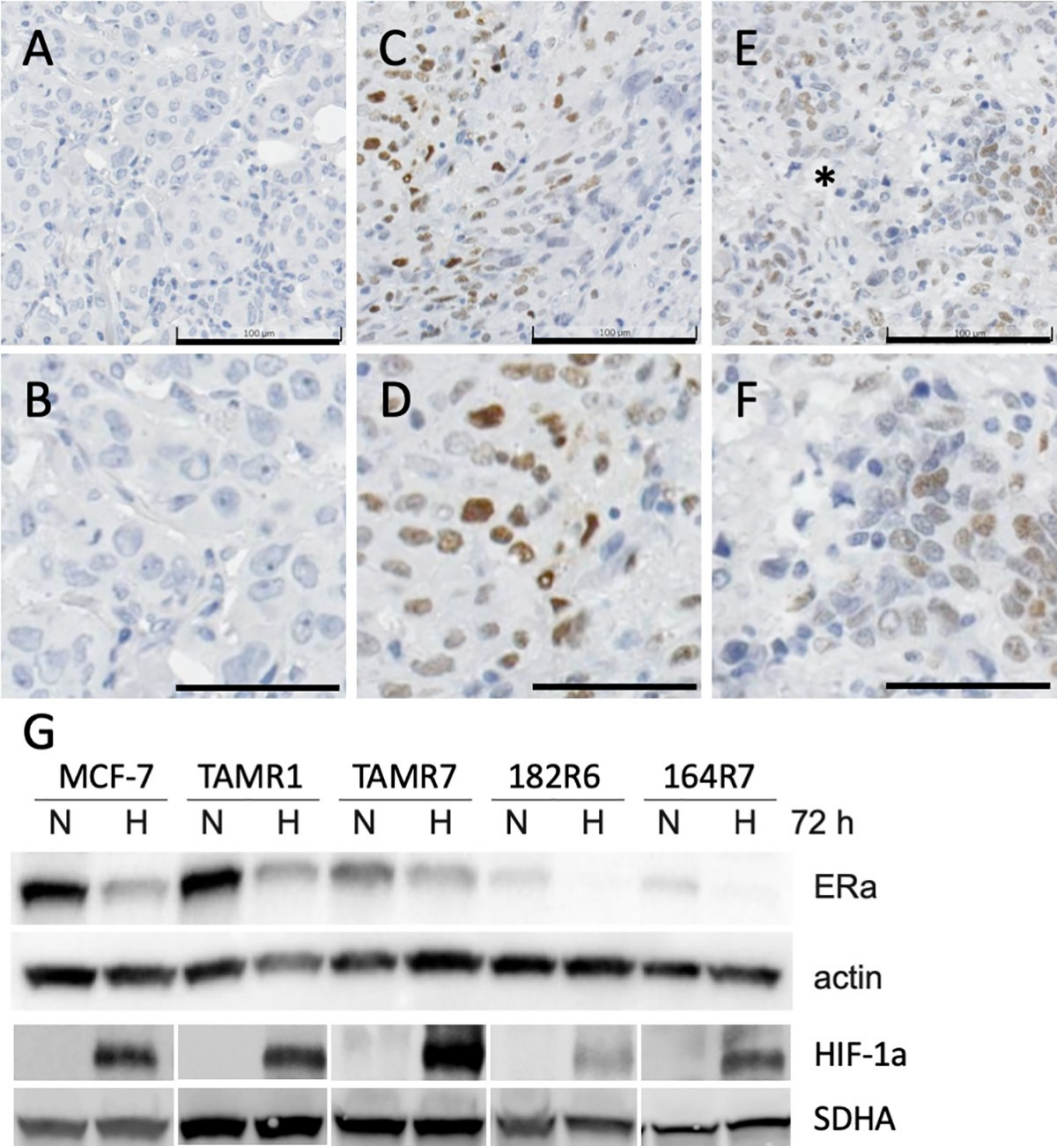
HIF-1α immunohistochemistry (IHC) and immunoblot. Human breast cancer sample examples from the contralateral breast cancer cohort negative (A, B) and positive (C-F) for HIF-1α IHC. * marks a necrotic area with perinecrotic cells staining positive for HIF-1α. A, C and E size bars 100 μm, B, D and F size bars 50 μm. Immunoblot analysis of ERα- and HIF-1α expression in MCF-7 breast cancer cells and in four derived strains with induced tamoxifen or fulvestrant resistance (G). Actin was used as loading control for ERα and SDHA for HIF-1α. In Fig 2D images have been spliced, as indicated by spacing between panels, to include all relevant data in one figure. The entire width of HIF-1α-immunoblots are shown in S1 Fig with places for splicing demarked with vertical black lines. Cells were cultured for 72 h at 21% (normoxia, N) or 1% (hypoxia, H) oxygen.

HIF-1α IHC-signal was exclusively nuclear in control samples of human cells cultured at hypoxic conditions (1% O_2_) as well as in patient breast cancer samples (Fig 2A–2F and S1A–S1C Fig). HIF-1α was undetectable in human breast cancer cells cultured in monolayer at normoxic conditions (21% O_2_) consistent with the instant degradation of HIF-1α in the presence of oxygen (S1 Fig) [25]. Vhl-deficient renal clear cell carcinoma cells, ROC-4, where HIF-1α protein is known to accumulate irrespective of oxygen conditions was used as a positive control for IHC and, as anticipated, we detected nuclear HIF-1α (S1 Fig).

**Fig 3:**
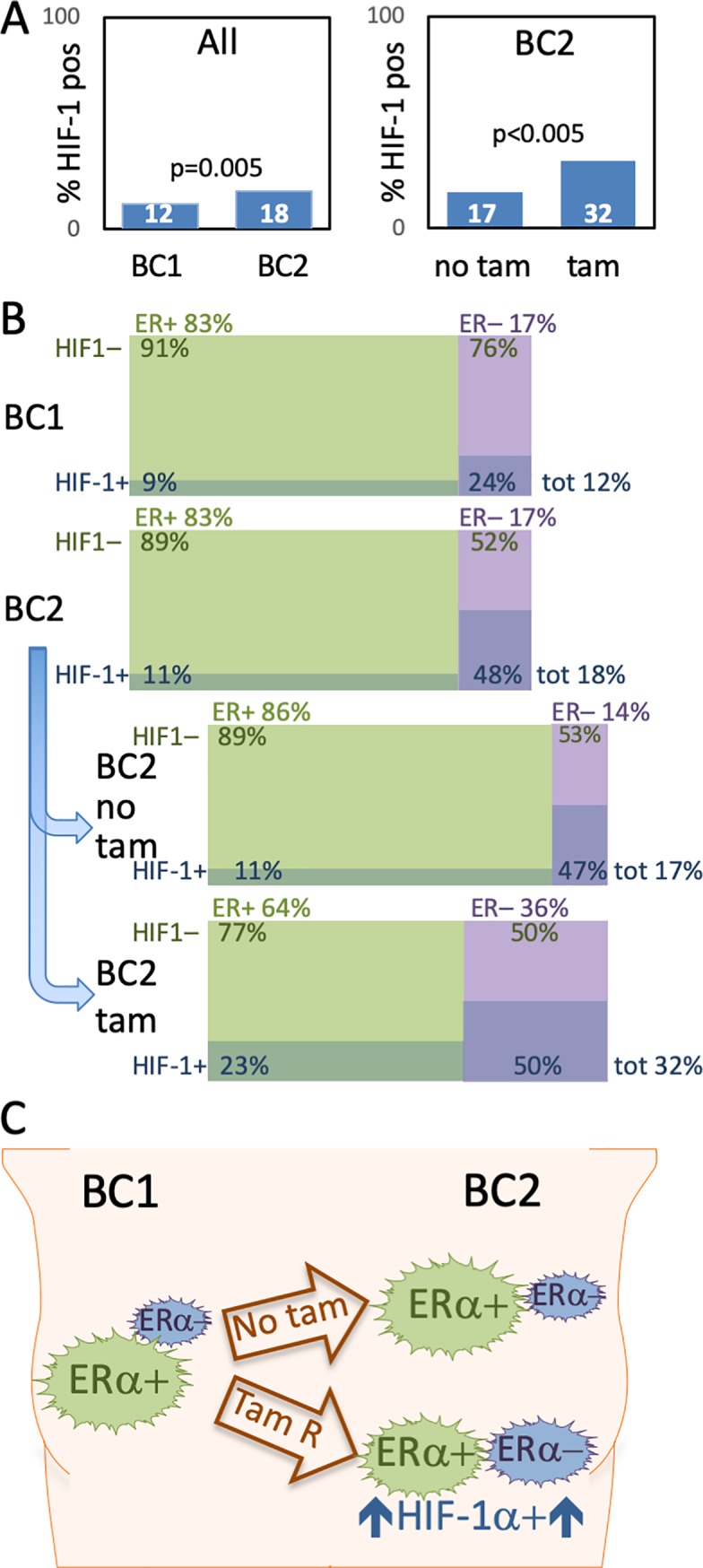
Fraction of HIF-1α-positivity in the contra lateral breast cancer material. Percentage of HIF-1α-positive tumors, in the entire material (left chart) and in BC2, with or without prior adjuvant tamoxifen (right chart) (A). Displaying the percentage of ERα-positive (ER+, > = 10% of cancer cells positive, green in charts), ERα-negative (ER–, < 10% of cancer cells positive, purple in charts), HIF-1α-positive (HIF-1α +, > = 1% of cancer cells positive, darker shade in charts) and HIF-1α negative (HIF-1α –, < 1% of cancer cells positive, lighter shade in chart) tumors. Data extracted from Tables 1 and 2. (B). Metachronous CBC (BC2) formed despite on-going tamoxifen treatment are more often HIF-1α positive than first tumors (BC1) and BC2 without prior tamoxifen. ER-negative tumors are also more frequent in the BC2-tumors diagnosed during on-going tamoxifen as is combined HIF-1α-positivity and ERα-negativity. Size of tumor symbol arbitrarily corresponds to phenotype frequency (C).

No consensus for the choice of cut-off value for HIF-1α was found in the literature. All tumors with HIF-1α signal above 1 in ≥1% of cell nuclei were considered HIF-1α-positive, corresponding to 12% of BC1 and 18% of BC2 (McNemars test p = 0.005) (Table 1, Fig 3A left panel). This increase in BC2 was to a large extent due to a doubling of the percentage of HIF-1α-positive tumors among the ERα-negative CBCs (24% vs. 48%, Tables 1, Fig 3B). The fraction of ERα-negative tumors was the same in BC1 and BC2 (17%, McNemars test p = 0.9, Table 1 and Fig 3B). In addition, there was a higher frequency of HIF-1α-positivity among the ERα-negative compared to ERα-positive tumors (Tables 1 and Fig 3B). This was seen when looking at BC1 (χ^2^-test: p<0.001), BC2 (χ^2^-test: p<0.001), as well as BC2 developed after tamoxifen treatment (χ^2^-test: p = 0.04). We did not see any association between the HIF-1α-status of BC1 and that of BC2 in the same patient (χ^2^-test: HIF-1α p = 0.3).

HIF-1α-positivity correlated to negative prognostic factors including low age at diagnosis, presence of lymph node metastasis, high tumor stage, ERα- and PR-negativity, overexpression of HER2, and high Ki67-expression (Table 1). In addition, HIF-1α-positivity was associated with a short time-interval to development of the BC2 (Table 1), which we previously showed to be associated with a shorter distant disease-free survival in CBC-patients [5]. In accordance with our previous findings in cultured breast cancer cells [15] we found a correlation between HIF-1α-positivity and EGFR-expression (Table 1, see below).

### Adjuvant tamoxifen increased HIF-1α-positivity in ERα-positive CBC

CBC diagnosed during adjuvant tamoxifen-treatment for BC1 (N = 60) was more often HIF-1α-positive than if no prior tamoxifen had been given (N = 522) (32% (18/56) *vs*. 17% (80/482) p<0.005) (Table 2, Fig 3A). Subgroup analyses showed an increased percentage of HIF-1α-positivity with prior tamoxifen in ERα-positive CBCs (N = 448) (23% (8/35) with prior tamoxifen *vs*. 11% (45/409) without prior treatment, Fig 3B). This increase was not seen in ERα-negative CBCs. The ERα-negative CBCs had a very high frequency of HIF-1α-positivity both with (50%) and without (47%) prior tamoxifen (Table 2, Fig 3B). However, adjuvant tamoxifen correlated to an increased fraction of ERα-negative CBC [18], which in turn had a high incidence of HIF-1α-positivity (Table 2, Fig 3B) and a worse outcome.

**Table 2:** HIF-1a, HIF-2a and EGFR-expression in BC2 in relation to prior tamoxifen.

N = 582^a^	HIF1 in BC2		P-value^b^	EGFR in BC2		P-value^b^
ER-status missing for 48, HIF1 for 44, and EGFR for 42 CBC.	Negative	Positive		Negative	Positive	
	N = 440 (82%)	N = 98 (18%)		N = 487 (90%)	N = 53 (10%)	
**All CBC**			0.004			0.8
No prior tamoxifen, N = 522	402 (83%)	80 (17%)		436 (90%)	47 (10%)	
Prior tamoxifen, N = 60	38 (68%)	18 (32%)		51 (89%)	6 (11%)	
*Missing*	*83*	*13*		*83*	*13*	
**ER-positive CBC**			0.04			0.2
No prior tamoxifen, N = 412	364 (89%)	45 (11%)		400 (98%)	9 (2%)	
Prior tamoxifen, N = 36	27 (77%)	8 (23%)		34 (94%)	2 (6%)	
*Missing*	*72*	*4*		*73*	*3*	
**ER-negative CBC**			0.8			0.007
No prior tamoxifen, N = 66	35 (53%)	31 (47%)		30 (45%)	36 (55%)	
Prior tamoxifen, N = 20	10 (50%)	10 (50%)		16 (80%)	4 (20%)	
*Missing*	*9*	*9*		*8*	*10*	

^a^ Patients with CBC during tamoxifen treatment *vs*. patients with no prior endocrine therapy included in analysis.

^b^ χ^2^-test

**Abbreviations:** BC1 f*irst breast cancer*, BC2 *second breast cancer*, CBC *Contralateral breast cancer*, EGFR *Epidermial growth factor receptor*, ER *Estrogen receptor*, HIF *Hypoxia-inducible factor*.

### Hypoxia affect ERα-expression

In line with the results above, we found that ERα is down-regulated, and HIF-1α levels increased, when ERα-positive breast cancer cells are exposed to hypoxia and HIF-1α accumulate (Fig 2G, S1 Fig). ERα-expressing breast cancer cells are generally sensitive to antiestrogens and respond to treatment with decreased proliferation and increased cell death. With prolonged exposure to antiestrogens resistant sub-lines have been generated from antiestrogen-sensitive breast cancer cell lines. Hypoxia-induced down-regulation of ERα was seen in the antiestrogen sensitive parental cells (MCF7), as well as in tamoxifen (TAMR1, TAMR7) and fulvestrant (182R6, 164R7) resistant cells (Fig 2G, S1 Fig). In fact, for the latter the threshold for ERα-detection with immunoblotting was approached.

### EGFR-expression correlates to HIF-1α-positivity and additional negative prognostic factors

In EGFR-positive tumors expression was confined to the cell membranes of cancer cells. EGFR expression was similar in BC1 and BC2 (13% vs. 10%, Table 1), with a strong correlation between EGFR-expression in BC1 and BC2 in the same patient (p<0.001).

HIF-1α-positivity correlated to EGFR-expression in patient breast cancer samples corroborating *in vitro* data that hypoxia and HIF-activity induce EGFR-expression. EGFR-expression was associated with negative prognostic factors including ERα/PR-negativity, HER2-overexpression, high tumor stage, high Ki67, low age at diagnosis, lymph node metastasis, and larger tumor size at diagnosis.

### Patients with HIF-1α-positive CBC-tumors have a worse prognosis

**Fig 4:**
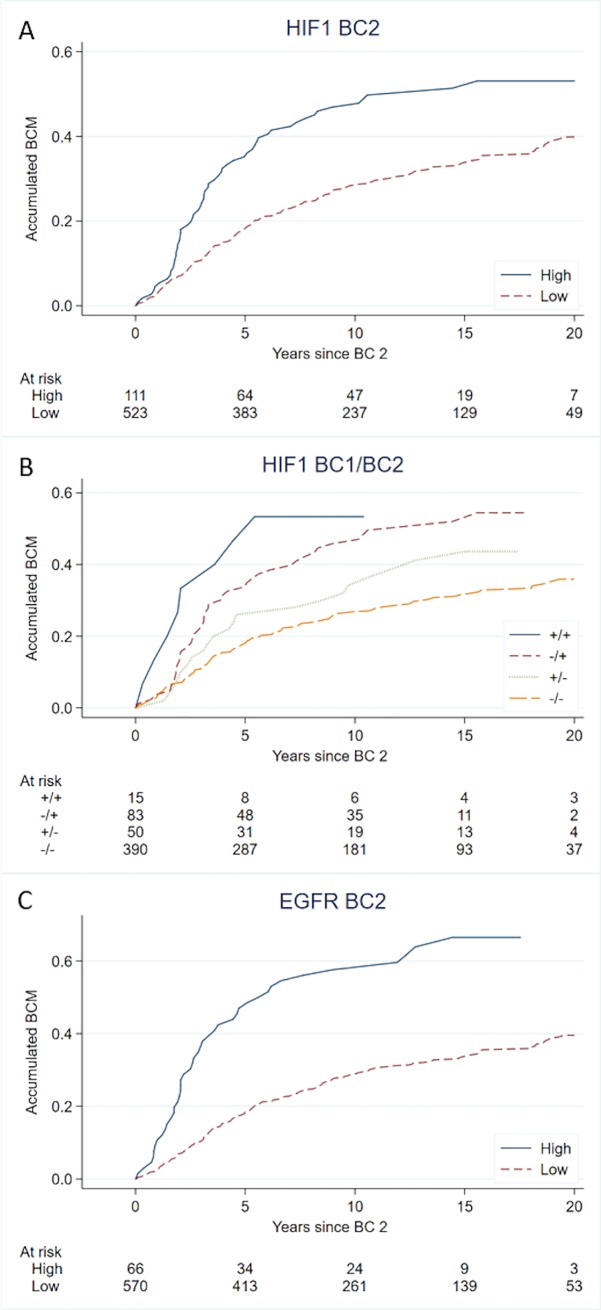
**Accumulating breast cancer mortality** (BCM) with HIF‐1α‐positivity in BC2 (A), HIF‐1α‐positivity in BC1 and BC2 (++ HIF-1α positive BC1+BC2, +- HIF-1α positive BC1 and negative BC2, -+ HIF-1α negative BC1 and positive BC2, -- HIF-1α negative BC1+BC2) (B), and with EGFR-expression (C).

Previous studies by our group showed that characteristics of BC2 have the highest influence on prognosis after development of CBC, although the characteristics of BC1 continue to have some prognostic impact [5, 18]. We find that HIF-1α-positivity in BC2 correlates to worse prognosis after CBC in both univariate and multivariate analyses (Table 3). Investigating HIF-1α-status in both BC1 and BC2 showed that patients with two HIF-1α-negative tumors had the lowest BCM and those with HIF-1α-positive BC2 the highest (Fig 4) (Cox-regression: BC1 _HIF1-_BC2_HIF1-_ reference, BC1_HIF1+_BC2_HIF1-_ HR 1.4 95%CI 0.91–2.3 p = 0.1, BC1_HIF1-_BC2_HIF1+_ HR 1.9 95%CI 1.3–2.6 p<0.001, BC1_HIF1+_BC2 _HIF1+_ HR 1.7 95%CI 0.83–3.5 p = 0.1).

**Table 3:** Breast Cancer Mortality after CBC in relation to HIF and EGFR-status of BC2.

	Univariable			Adjusted for BC1^a^			Adjusted for BC1, BC2 and treatment^b^		
	HR	95% CI	p-value	HR	95% CI	p-value	HR	95% CI	p-value
**HIF1 in BC2 (High vs Low)**									
All patients (N = 634)	1.6	1.2–2.2	0.001	1.7	1.2–2.4	0.004	1.6	1.0–2.5	0.03
*ER-positive CBC (N = 520)*	*1*.*5*	*0*.*96–2*.*2*	*0*.*08*	*1*.*3*	*0*.*80–2*.*2*	*0*.*3*	*1*.*3*	*0*.*72–2*.*3*	*0*.*4*
*ER-negative CBC (N = 104)*	*1*.*1*	*0*.*69–1*.*9*	*0*.*6*	*1*.*4*	*0*.*78–2*.*7*	*0*.*2*	*2*.*6*	*1*.*1–6*.*4*	*0*.*04*
**EGFR in BC2 (High vs Low)**									
All patients (N = 636)	2.4	1.7–3.3	<0.001	2.2	1.5–3.3	<0.001	1.1	0.59–2.0	0.8
*ER-positive CBC (N = 521)*	*2*.*7*	*1*.*5–5*.*2*	*0*.*002*	*2*.*1*	*0*.*95–4*.*6*	*0*.*07*	*1*.*3*	*0*.*49–3*.*4*	*0*.*6*
*ER-negative CBC (N = 104)*	*1*.*2*	*0*.*75–2*.*0*	*0*.*4*	*1*.*4*	*0*.*74–2*.*8*	*0*.*3*	*0*.*91*	*0*.*40–2*.*3*	*0*.*8*

^a^ Adjusted for calendar-period of diagnosis, time-interval between tumors, age, and characteristics of BC1 (lgl-metastasis, size, ER, HER2, Ki67). Subgroup analyses regarding ER-status of course not adjusted for ER-BC2.

^b^ Multivariable analyses adjusted for calendar-period of diagnosis, time-interval between tumors, age, and characteristics and treatment given for BC1 and BC2 (lgl-metastasis, size, ER, HER2, Ki67, radiotherapy, chemotherapy, and tamoxifen). Subgroup analyses regarding ER-status of course not adjusted for ER-BC2.

A high EGFR-expression in BC2 was associated with worse BCM in univariate and multivariate analyzes adjusted for characteristics of BC1 (Fig 4, Table 3). Subgroup analyses suggested this effect to be strongest in ERα-positive CBC (Interaction EGFR/ERα-status in relation to prognosis, p = 0.06).

## Discussion

We have explored the status of HIF-1α and EGFR in a unique cohort of CBC-patients that, to our knowledge, is the largest of its kind with access to detailed patient-information and a long follow-up period along with tumor-tissue samples. The expression of hormone receptors, HER2, and Ki67 was analyzed in the same samples [18]. This material is a human *in vivo*-model of therapy-resistance since, by definition, a second tumor developed during treatment is resistant to the given treatment, making this material uniquely suitable for exploring mechanisms of therapy-escape.

Endocrine treatment plays an important role in both adjuvant and metastatic breast cancer therapy improving survival for many patients. Development of treatment-resistance is a great challenge; with many patients relapsing despite adjuvant therapy and in metastatic disease most tumors eventually become resistant. In CBCs developed during adjuvant tamoxifen we found a significantly increased proportion of HIF-1α-positive tumors compared to CBCs in patients with no prior tamoxifen treatment. A novel finding in a unique and large material of clinical samples of breast cancers diagnosed during adjuvant tamoxifen therapy. We further show that HIF-1α-positive CBC-tumors were associated with a worse tumor phenotype and a shorter patient survival (Fig 4), corroborating previous data linking HIF-1α to a worse outcome in breast cancer [13, 26]. Taken together our findings suggest a role for HIF-1α in the development of antiestrogen treatment-resistance and progression into a more aggressive tumor phenotype.

We also found a strong correlation between HIF-1α-positivity and EGFR-expression in both primary tumors and CBCs, strengthening our previously reported finding that hypoxia/HIF and EGFR cross-talk in tamoxifen-resistance [15]. This finding is promising since EGFR is a drug-able target with existing small molecule inhibitors (gefitinib, erlotinib) and inhibiting monoclonal antibodies (cetuximab) approved for cancer treatment. Increased knowledge about the signaling factors and pathways leading to treatment escape could aid in finding new drug-able targets, but also in making widened use of medications already available. Finding out whether the effect of HIF-1α on tamoxifen-sensitivity and breast cancer survival is depending on down-stream EGFR-signaling is key in this respect. Increased knowledge in this field can lead to individualized treatment customized to proliferation mechanisms active in each individual tumor. In addition, strategies to minimize tumor hypoxia may contribute to better outcome of cancer therapy.

The increased fraction of HIF-1α-positive tumors with adjuvant tamoxifen was confined to ERα-expressing tumors. However, there was a concurrent increase in the proportion of ERα-negative tumors [19], which had a high percentage of HIF-1α-positivity irrespective of adjuvant tamoxifen. In the BC1 tumors as well as in CBCs we found a higher incidence of HIF-1α-positivity among ERα-negative tumors (Table 1, Table 2 and Fig 3). Previous data on correlation between HIF-1α and ERα-expression are diverging. In one previous article based on 100 breast tissue samples, of which 40 represented invasive cancer, HIF-1α-positivity was on the contrary reported to be associated with a high ERα-expression [27], whereas the same group detected a weak correlation between HIF-1α and loss of ERα-expression in a subsequent breast cancer material encompassing 150 patients with stage I-II tumors [13]. A later study of almost 200 patient samples, reported no significant correlation between tumor HIF-1α and ERα status [26]. The present data is, to our knowledge, the largest cohort so far (including >1200 invasive breast tumors) in which correlation between HIF-1α and ERα status has been evaluated.

One explanation of the more frequent HIF-1α-positivity in ERα-negative breast cancer could be that these tumors have a high proliferation rate, leading to hypoxic foci. Furthermore, we show that hypoxia induce down-regulation of ERα expression in breast cancer cells (in antiestrogen-resistant cells even approaching the threshold of detection), corroborating our previous data from DCIS lesions where ERα-expression was lost in HIF-1α-positive perinecrotic cells [28]. We cannot determine from the current CBC material whether the increased frequency of ERα-negative tumors after adjuvant tamoxifen is due to potential ERα-positive lesions deteriorating at an early stage or if the developing lesions down-regulate their ERα-expression. Presumably both alternatives are possible. From our current and previous findings, we suggest that one mechanism for down-regulation of ERα-expression and escape of antiestrogen therapy could be hypoxia/HIF-dependent down-regulation of the ERα-expression. Hence, another explanation for the higher percentage of HIF-1α-positivity among ERα-negative tumors may be that hypoxia/HIF actually induce this phenotype.

HIF-1α-positive tumors increased from 24% in ERα-negative BC1 to 47% in ERα-negative BC2. This higher frequency of HIF-1α-positivity was not related to tamoxifen exposure, since the percentage was similar in ERα-negative CBC developed with/without previous tamoxifen. Instead, our most plausible explanation is that patients with an aggressive BC1 more often experienced disease relapse (both ERα-negativity and HIF-1α-positivity are associated with a worse prognosis) before development of a CBC, and hence were excluded from this study.

## Conclusions

We show that tamoxifen resistant CBCs, *i*.*e*. developed while under tamoxifen therapy, are more often HIF-1α-positive compared to CBCs in patients that never received antiestrogens. Present and previous data from our group as well as others suggest that hypoxia/HIF-activity induce EGFR and down-regulate ERα-expression, both suggested mechanisms for endocrine therapy resistance. In addition, we find that HIF-1α correlates to several other negative prognostic factors and to shorter survival. Taken together this suggests important roles for HIF-1α in tumor progression and development of antiestrogen treatment escape mechanisms. That the effects of hypoxia and HIF-1α on therapy resistance and survival at least in part is in cross-talk with EGFR opens for new application of established EGFR-inhibitors as well as for emerging HIF-inhibitors combined with strategies to minimize tumor hypoxia.

## Supporting information

S1 Fig HIF-1α immunohistochemistry (IHC) and immunoblot. T47D breast cancer cells grown at 21% (A) and at 1% oxygen (B). Human clear cell renal carcinoma cells, ROC4, with Vhl-mutation leading to nuclear HIF-1α accumulation at 21% oxygen (C). 40 x, size bars 20 μm. Immunoblots from Fig 2 showing the entire gel-width. No manipulation of signal intensity or esolution were performed (neither in panels in Fig 2). DIP, positive control for HIF-1a accumulation at normoxia with addition of 100 μM 2,2’-dipyridyl (Sigma) to the cell culture medium (D-H). Framed lanes are shown in Fig 2.
(TIF)

## Abbreviations

BC1: first breast cancer
BC2: second breast cancer
BCM: breast cancer mortality
CBC: contralateral breast cancer
CI: confidence interval
EGFR: epidermal growth factor receptor
ERα: estrogen receptor α
FCS: fetal calf serum
FFPE: formalin-fixed paraffin-embedded
HIF: hypoxia-inducible factor
HR: hazard ratio
HRE: hypoxia responsive element
IHC: immunohistochemistry
PR: progesterone receptor
TMA: tissue micro-array
