# Supplementary figures and images for: Expression of HIF-1α is related to a poor prognosis and tamoxifen resistance in contralateral breast cancer
Source: PLoS One. 2019 Dec 10;14(12):e0226150. doi: 10.1371/journal.pone.0226150 (PMC6903737; doi:10.1371/journal.pone.0226150)

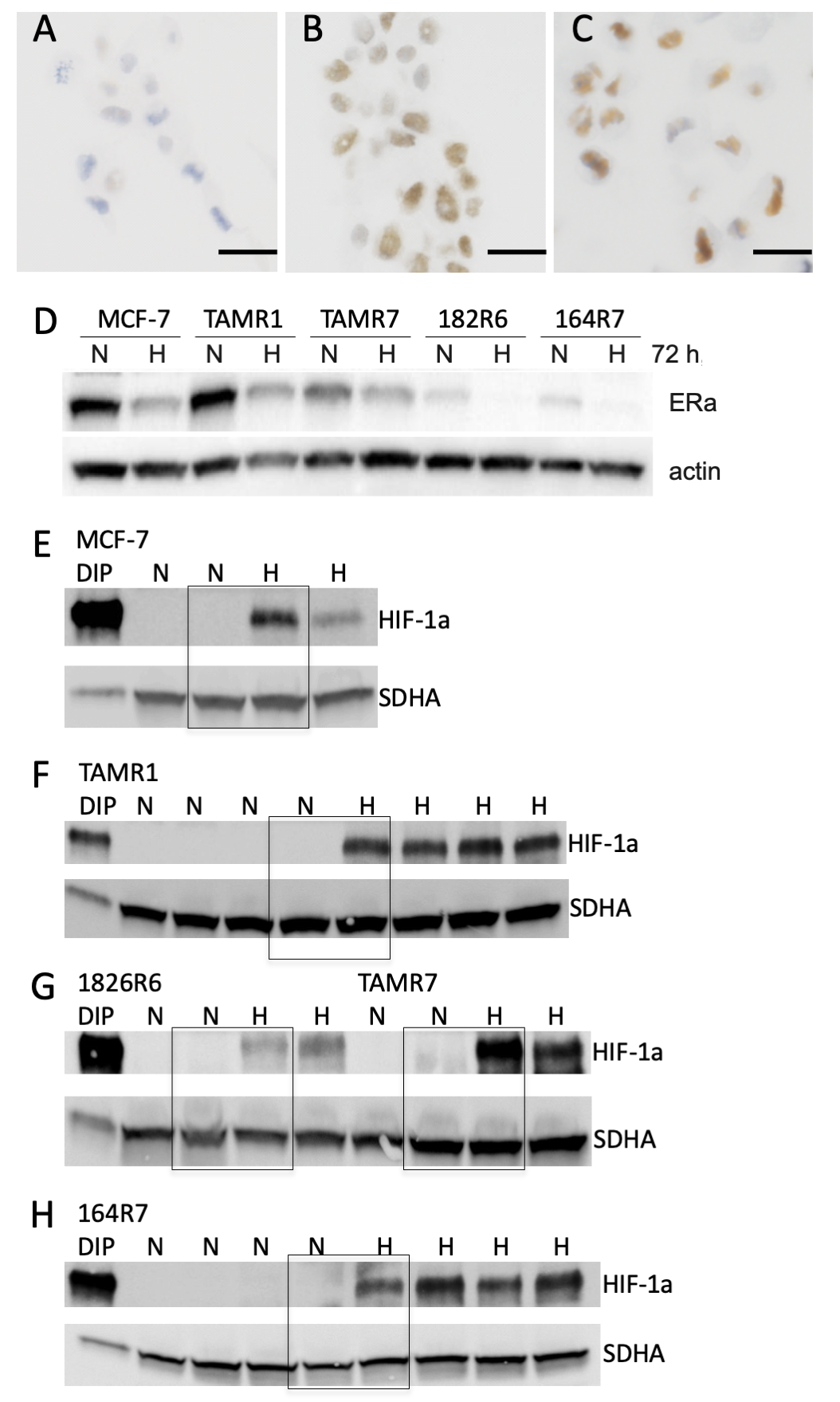

Supplement: S1 Fig — T47D breast cancer cells grown at 21% (A) and at 1% oxygen (B). Human clear cell renal carcinoma cells, ROC4, with Vhl-mutation leading to nuclear HIF-1α accumulation at 21% oxygen (C). 40 x, size bars 20 μm. Immunoblots from Fig 2 showing the entire gel-width. No manipulation of signal intensity or esolution were performed (neither in panels in Fig 2). DIP, positive control for HIF-1a accumulation at normoxia with addition of 100 μM 2,2’-dipyridyl (Sigma) to the cell culture medium (D-H). Framed lanes are shown in Fig 2. (TIF) [file pone.0226150.s001.tif]
